# Transcription Factors in the Cellular Response to Charged Particle Exposure

**DOI:** 10.3389/fonc.2016.00061

**Published:** 2016-03-21

**Authors:** Christine E. Hellweg, Luis F. Spitta, Bernd Henschenmacher, Sebastian Diegeler, Christa Baumstark-Khan

**Affiliations:** ^1^Cellular Biodiagnostics, Department of Radiation Biology, Institute of Aerospace Medicine, German Aerospace Centre (DLR), Cologne, Germany

**Keywords:** charged particles, p53, Nrf2, NF-κB, AP-1, Sp1, CREB, EGR-1

## Abstract

Charged particles, such as carbon ions, bear the promise of a more effective cancer therapy. In human spaceflight, exposure to charged particles represents an important risk factor for chronic and late effects such as cancer. Biological effects elicited by charged particle exposure depend on their characteristics, e.g., on linear energy transfer (LET). For diverse outcomes (cell death, mutation, transformation, and cell-cycle arrest), an LET dependency of the effect size was observed. These outcomes result from activation of a complex network of signaling pathways in the DNA damage response, which result in cell-protective (DNA repair and cell-cycle arrest) or cell-destructive (cell death) reactions. Triggering of these pathways converges among others in the activation of transcription factors, such as p53, nuclear factor κB (NF-κB), activated protein 1 (AP-1), nuclear erythroid-derived 2-related factor 2 (Nrf2), and cAMP responsive element binding protein (CREB). Depending on dose, radiation quality, and tissue, p53 induces apoptosis or cell-cycle arrest. In low LET radiation therapy, p53 mutations are often associated with therapy resistance, while the outcome of carbon ion therapy seems to be independent of the tumor’s p53 status. NF-κB is a central transcription factor in the immune system and exhibits pro-survival effects. Both p53 and NF-κB are activated after ionizing radiation exposure in an ataxia telangiectasia mutated (ATM)-dependent manner. The NF-κB activation was shown to strongly depend on charged particles’ LET, with a maximal activation in the LET range of 90–300 keV/μm. AP-1 controls proliferation, senescence, differentiation, and apoptosis. Nrf2 can induce cellular antioxidant defense systems, CREB might also be involved in survival responses. The extent of activation of these transcription factors by charged particles and their interaction in the cellular radiation response greatly influences the destiny of the irradiated and also neighboring cells in the bystander effect.

## Introduction

Understanding the cellular radiation response is an essential prerequisite for improving cancer radiotherapy, including carbon ion therapy. The same holds true for the risk assessment of astronauts’ and for effective countermeasure development.

Radiotherapy of cancer with protons and carbon ions profits from a more precise dose deposition with charged particle beams in the tumor and in the case of carbon ions, also a higher biological effectiveness in cell killing compared to conventional radiotherapy. There are hints that with carbon ions, cell killing is less dependent on factors such as oxygen concentration and alterations in cellular signaling pathways such as the p53 pathway.

Astronauts on exploration missions are subjected to not only greater amounts of natural radiation in space than they receive on Earth but also to a differing radiation quality, which can result in immediate and long-term risks. Besides protons and α-particles, heavier nuclei are part of the radiation field encountered in space. Heavy ions represent an important part of galactic cosmic rays because of their high biological effectiveness (1).

The radiation quality of energetic ions, including protons, α-particles, and heavy ions, is usually characterized by the linear energy transfer (LET) in matter which is a measure of the average energy transferred when an ionizing particle passes through matter and loses energy (2). Indirectly, it gives information about the ionization density along the particle track.

In the cellular response to radiation, several sensors detect the induced DNA damage and trigger signal transduction pathways, resulting in cell death or survival with or without mutations (3, 4). The activation of several signal transduction pathways by ionizing radiation (IR) results in altered expression of series of target genes. The promoters or enhancers of these genes may contain binding sites for one or more transcription factors, and a specific transcription factor can influence the transcription of multiple genes. A meta-analysis revealed that two p53-dependent genes, GADD45 (especially GADD45α) and CDKN1A, and genes associated with the NER pathway (e.g., XPC) are consistently upregulated by IR exposure (5). Importantly, the transcribed subset of target genes is critical for the decision between resuming normal function after cell-cycle arrest and DNA repair, entering senescence, or proceeding through apoptosis in cases of severe DNA damage (5) and thereby for the cellular destiny and for the outcome of cancer radiotherapy. The changes in gene expression induced by IR *via* transcription factors depend on dose, dose rate, time after irradiation, radiation quality, cell type, inherited or accumulated mutations in signaling pathways, cell-cycle phase, and possibly on other factors (Table 1). Twelve years ago, the transcription factors to be activated after exposure to clinically relevant doses of IR were summarized, resulting in the short list of p53, nuclear factor κB (NF-κB), and the specificity protein 1 (SP1)-related retinoblastoma control proteins (RCPs) (6). In this review, the role of transcription factors in the cellular response to IR is summarized with a special focus on charged particles as far as data are available.

**Table 1:** **Transcription factor activation by ionizing radiation**.

Experimental model	Radiation quality	Dose	Method	Effect	Reference
**p53**					
H1299 (originally p53 null)	X-rays	2–5 Gy	Colony-forming ability (CFA) assay, acridine orange/ethidium bromide staining, Western blot, quantitative real time RT-PCR (RT-qPCR)	Wildtype p53 cells: higher sensitivity compared to p53 null or mutated p53 cells	(172)
	^12^C 290 MeV/u	2–5 Gy		Low LET radiation ⇑ p53-dependent apoptosis	
				High LET ⇑ p53-independent apoptosis	
A549, AGS, and MCF-7	X-rays	0–12 Gy	RT-qPCR, Western blot, flow cytometry, luciferase reporter assay, CFA assay	miR-375 overexpression ⇑ p53 expressions ⇓	(173)
	Etoposide	0–100 μM		Radiosensitivity ⇓	
HCT116 (colorectal cells) p53 wt and ko cells	X-rays	0–8 Gy	Viability assay (transwell co-culture), micronuclei and apoptosis evaluation, beta-galactosidase staining, RT-qPCR	Low doses: no difference between cell lines	(174)
				Higher doses: significant differences, e.g., micronuclei ⇑ and apoptotic cells ⇑ in p53^−/−^ cells, p53^+/+^: high levels of senescence	
Lung epithelial cells	α-particles (^238^Pu) X-rays	0–1.2 Gy 0–2.5 Gy	Flow cytometry	p53 expression levels ⇑	(175)
HCT116	^12^C 290 MeV/u	0–3 Gy	CFA assay, flow cytometry, iimmunofluorescence	X-rays ⇑ higher sensitivity and apoptosis ⇑ in p53^+/+^ cells	(176)
	X-rays	0–8 Gy		C-ions ⇑ no difference of sensitivity (mitotic catastrophe ⇑ in p53^−/−^ cells, apoptosis ⇑ in p53^+/+^ cells)	
**NF-κB**					
Human, mouse, rat, hamster normal, transformed and tumor cell lines and primary cells, animal models (rat, mouse)	X-rays, γ-radiation, protons, α-particles, Fe ions, C ions, Ar ions	0.05–100 Gy	EMSA, Western blot, immunofluorescence, reporter assays, oligonucleotide enzyme-linked immunosorbent assay (ELISA)	Dose, cell line/cell type, and radiation quality-dependent activation	(70)
**Nrf2**					
NIH-3T3 MCF7-AREc32 Embryonic fibroblasts from wt and Nrf2 ko mice	γ-radiation^137^Cs source	2–8 Gy, 10 Gy	Luciferase assay, RT-qPCR, Western blot, CFA assay, ROS measurement (H_2_DCFH-DA)	No short-term activation of Nrf2 activation Late activation of Nrf2 Nrf2 activation after fractionated irradiation	(88)
PC3 and DU145 prostate cancer cell lines	γ-radiation^60^Co source	1–10 Gy	Electrophoretic mobility shift assay (EMSA), RT-qPCR	Differences in basal Nrf2 expression determine resistance to irradiation	(99)
		4 and 8 Gy	Knockdown (kd) of Nrf2 and heme oxygenase-1 (HO-1) expression using short hairpin RNA (shRNA)	High basal Nrf2 activity ⇑ Nrf2 activity ⇑, target gene expression ⇑ (DU145 cells) ⇑ higher radioresistance than PC3 cells	
				Knockdown of Nrf2 ⇑ cell death ⇑	
Murine T-cell lymphoma cell line EL-4	γ-radiation ^60^Co source	4 Gy	shRNA-kd, RT-qPCR, EMSA	ERK and Nrf2 interact in radioresistance of EL-4 cells	(102)
Dermal fibroblasts from wt mice and Nrf2 and Keap1-KO mice	UV-A-UV-B radiation	10.000 mJ/cm^2^	Western blot, immunofluorescence, flow cytometry	UV-A, but not UV-B, induces Nrf2 activity, cellular survival depends on Nrf2	(103)
C57BL/6, CD-1, and SJL/C57BL/6 CD45.1 mice	γ-radiation	6.9, 7.0, 7.1, 7.25, 7.3, 10 Gy TBI	RT-qPCR	Interplay between Nrf2 and Notch signaling, Nrf2 mediates Notch signaling and increases hematopoietic stem cell function	(106)
*Mx-Cre-Keap1^flox/flox^*	^137^Cs source				
*CMVCre-Keap1^flox/flox^*	^12^C^6+^ ions	2 Gy	RT-qPCR of Nrf2 downstream genes NAD(P)H quinine oxidoreductase 1 (NQO1), HO-1, gamma-glutamyl cysteine synthetase (γ-GCS), immunofluorescence, Western blot	NQO1, HO-1, γ-GCS ⇑ in curcumin-pretreated mice	(108)
*Keap1^flox/flox^* mice					
HCEC CT7s cells (immortalized colon epithelic cells)	γ-radiation ^137^Cs source (cells)	4–5 Gy	Immunohistochemistry, Western blot, subcellular fractionation, immunofluorescence, assay for chromosomal aberrations at metaphase, shRNA against Nrf2, DNA fiber assay, ChIP qPCR	Nrf2 enhances DDR and reduces number of DNA DSB	(109)
C57BL/6 wt mice	X-rays (mice)	7.5–10 Gy TBI		Nrf2 ⇑ 53BP1 expression ⇑	
EA.hy926 and HMVEC cells	Photons from linear accelerator	0, 0.3, 0.5, 0.7, 1 Gy	RT-qPCR, flow cytometry, Western blot, enzyme activity of glutathione peroxidase, EMSA	Non-linear activation of Nrf2 and target genes	(107)
				Nrf2 activation prior to irradiation ⇑ cell adhesion ⇑	
				Nrf2 expression and binding to DNA lowest at 0.5 Gy	
**CREB**					
Human U1-Mel cell line	^60^Co γ-rays	4.5 Gy	EMSA with nuclear extracts	CREB DNA binding ⇑	(123)
Jurkat leukemic T cell line	10 MV X-rays	1.5 and 6 Gy	Western blot	CREB phosphorylation ⇑	(115)
K562 erythroleukemia cells	10 MV X-rays	1.5 and 15 Gy	Western blot	CREB phosphorylation ⇑	(119)
Chinese Hamster V79 cells	^12^C^5+^ ions	0.1 and 1 Gy	Western blot	p44/42 MAPK ⇑	(125)
AG1522 human diploid skin fibroblasts	α-particles (^238^Pu source)	0.01, 0.05, and 0.10 Gy	Western blot	p38 MAPK and ERK 1/2 ⇑	(126)
**AP-1**					
AG1522 human diploid skin fibroblast	α-particles (^238^Pu source)	0.003 and 0.006 Gy	EMSA	AP-1 DNA-binding activity ⇑	(126)
MRC5CV1 normal human fibroblasts	^137^Cs γ-rays	20 Gy	Western blot	c-jun phosphorylation ⇑	(131)
			EMSA	AP-1 DNA-binding activity ⇑	
ROS 17/2.8 osteoblasts	X-rays	5 Gy	EMSA with supershift	AP-1 DNA-binding activity ⇑	(132)
Spontaneously immortalized human breast epithelial cell line MCF-10F	α-particles, LET 150 keV/μm	6 and 1.2 Gy	Northern blot and immunochemical protein staining	c-jun, c-fos, FRA1 RNA, and protein expression ⇑	(135)
C57BL/6J mice	^56^Fe ions, 1000 MeV/n, LET 148 keV/μm	1.6 Gy	SOD 1/2 and catalase activity, NADPH oxidase activity assay and immunohistochemistry of p-H3	SOD 1/2, catalase, NADPH oxidase and mitogenic activity ⇑	(98)
**Sp1**					
Normal human diploid fibroblasts	6 MV X-rays	0.5, 2.5, 5, 10, 20, 40 Gy	Western blot	Sp1 expression and phosphorylation ⇑	(141)
U1-Mel cells	^137^Cs γ-rays	3 and 4.5 Gy	EMSA and Western blot	Sp1 DNA binding and phosphorylation ⇑	(142)
H1299	α-particles, LET 123 keV/μm	1 Gy	IPA upstream regulator analysis	Sp1 network involvement	(145)
Normal human fibroblasts (HFL 3)	C ions, 290 MeV/n, LET 70 keV/μm	2 Gy	PCC assay and immunofluorescence	DNA-PKc autophosphorylation ⇑	(146)
	Fe ions, 500 MeV/n, LET 200 keV/μm				
**EGR-1**					
Isolated lymphocytes	Na ^211^At α-particles	0.05–1.6 Gy	RT-qPCR	EGR-1 gene expression ⇑	(134)
Prostate cancer cells PC-3	100 kV X-rays	5 Gy	Western blot	Protein induction ⇑	(147)
Human HL 525 myeloid leukemia cells	^137^Cs γ-rays	20 Gy	Western blot	Protein expression ⇑	(148)

## p53

The transcription factor *TP53* (*p53*) was first described in 1979 (7), and many names have been attributed to the factor that belongs to the class of tumor suppressor genes. The transcription factor was called “an acrobat in tumorigenesis” (8), the “good and bad cop” (9), a “death star” (10), and even the “guardian of the genome” (11). p53 is involved in the regulation of cellular survival, immune responses, and inflammation, resulting in eminent importance in cancerogenesis and inflammation. Nowadays, it is known that defects in p53 are directly or indirectly involved in the majority (>50%) of human cancers as described by the International Cancer Genome Consortium (ICGC).

The human p53 gene is located on the short arm of chromosome 17 (17p13) and the protein size is 393 amino acids (~43 kDa). It is composed of several domains: the N-terminus contains a transactivation domain for downstream gene activation (1–43). A proline rich domain follows that mediates the response to DNA damage through apoptosis (58–101). The DNA-binding region or domain (DBD) is next (102–292) followed by an oligomerization domain (320–355) that interacts with other p53 monomers (p53 is capable of tetramerize). The C-terminus (356–393) is leucine rich and contains three putative nuclear localization signals (NLS) and so-called nuclear export signals (NES). It is postulated that when oligomerization occurs, NES are masked and p53 is retained in the nucleus. The DBD is the core domain, and it is composed of a variety of structural motifs. Single mutations within this domain can cause a major conformational change. There are in total 12 isoforms of p53 in humans discovered until now (12).

**Figure 1:**
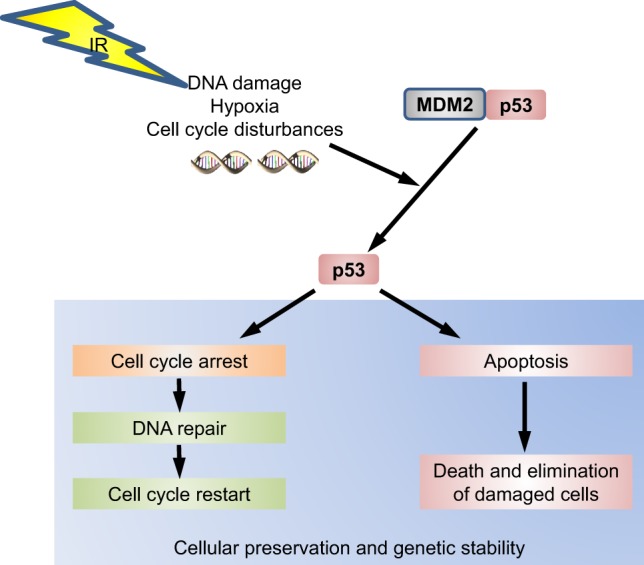
**The p53 pathway**. p53 is under normal conditions inactivated by murine double minute 2 (MDM2). When, e.g., DNA damage occurs, p53 dissociates from its regulatory MDM2 complex by various pathways. In this active state, phosphorylated p53 will induce a cell-cycle arrest to permit either repair and therefore survival of the cell or induce apoptosis to eliminate a damaged cell.

p53 has been recognized as an important checkpoint protein in the DNA damage response (DDR), which transcriptionally controls target genes involved in multiple response pathways that are as diverse as cell-cycle arrest and survival or death by apoptosis (13). It is thereby important for explaining the diversity of cellular responses to IR exposure. p53 has a short half-life and is stabilized in response to a variety of cellular stresses after phosphorylation by ataxia telangiectasia mutated (ATM) (13). After exposure to IR, phosphorylation of the serine residues 15 and 20 on p53 by checkpoint kinase 2 (CHK2) reduces its binding to MDM2, which in its bound state targets p53 for degradation by the proteasome pathway (Figure 1). Thus, dissociation of p53 from MDM2 prolongs the half-life of p53 (14). Other proteins, such as Pin 1, Parc, and p300, and p300/CBP-associated factor (PCAF) histone acetyltransferases regulate the transactivation activity of p53 (13). For efficient repair, especially in non-dividing cells, cellular levels of deoxyribonucleotides are increased during the DDR by p53-dependent transcriptional induction of the ribonucleotide reductase RRM2B (p53R2) (15).

It is accepted that the severity of DNA damage is the critical factor in directing the signaling cascade toward reversible cell-cycle arrest or apoptosis (13, 15). As part of the signaling cascade, the abundance of p53 protein, specific posttranslational modifications, and its interaction with downstream effectors, such as GADD45α or p21, may be responsible for directing the cellular response at this decision point (14).

Recently, Gudkov and Komarova (16) proposed that after total body irradiation (TBI) of mice severe damage occurs in tissues prone to p53-dependent apoptosis [the apoptosis response of p53 after X-irradiation was already shown in murine experiments 1996 by Norimura et al. (17)], such as the hematopoietic system, hair follicles, and oligodendroblasts in the spinal cord. Other tissues, such as the vascular endothelial cells (ECs) of the small intestine react to p53 activation by cell-cycle arrest and activation of DNA repair (16). Connective tissues and epithelial cells usually respond with growth arrest to p53 activation (16). The authors conclude from animal models that p53 is the key component of the toxicity of IR or radiomimetic (DNA damaging) drugs. It thereby contributes to the hematopoietic component of the acute radiation syndrome and leads to severe adverse effects of cancer treatment (16).

p53-dependent GADD45α upregulation may play a role in apoptosis by activating the c-Jun N-terminal kinase (JNK) and/or p38 mitogen-activated protein kinase (MAPK) signaling pathways (18). Besides GADD45α, p53 regulates the expression of other proteins involved in apoptosis, including membrane-bound proteins, such as Fas/CD95, TP53 apoptosis effector related to PMP22 (PERP), and KILLER/DR5, cytoplasm-localized proteins, such as p53-inducible death domain-containing protein (PIDD) and PIGs, and mitochondrial proteins, such as BAX, NOXA, PUMA, p53Aip1, and BID (13, 14). The induction of these pro-apoptotic genes seems to be tissue specific (13). p53 also directly interacts with BAX, BCL-XL, and BCL-2 at the mitochondrial membrane (14).

So far, p53 plays a crucial role in the cellular radiation response. In future, the treatment of patients suffering from cancer will be personalized; this means that the combination of cytostatic agents and radiotherapy has to be individualized also depending on the tissue affected. For colorectal carcinoma cell lines, many tests are being performed with distinct agents where, e.g., gemcitabine, paclitaxel, or irinotecan are used in order to optimize the treatment results in combination with carbon ions. It has been already shown that after C-12 ion irradiation in cells lacking p53, paclitaxel and gemcitabine were very effective as well as irinotecan on p53 wild-type (wt) cells (19). Nevertheless, a problem with targeting the p53 pathway as a helping tool in cancer therapy by activation and thereby unleashing the protective attitudes of this pathway is that in hematological malignancies there is a low incidence of p53 mutations. Here, maybe MDM2 proteins can be addressed.

The bystander effect is a field that still needs to be understood and where experiments and the effects of a possible p53 response are barely recognized. First observations though, show that in mammalian cells lacking p53 in comparison to wt p53, the cells respond upon heavy ion exposure in the already known ways when directly irradiated (20). This fact is also to be taken into consideration when irradiation of patients is to be performed even though it does not play a major role, since the main goal is still targeting and eliminating the malignant tumors in an efficient manner.

**Figure 2:**
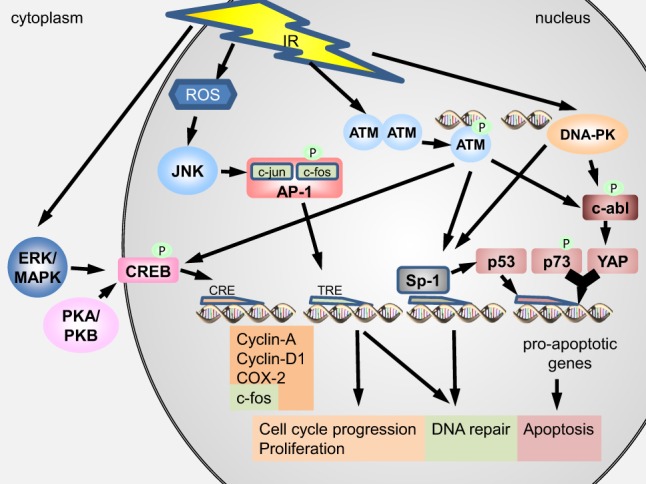
**Activation of the transcription factors CREB, AP-1, SP1, p73, and YAP upon irradiation ionizing radiation (IR) can activate protein kinase A (PKA) and B (PKB) as well as ERK/MAPK in the cytoplasm**. Exposure to IR can produce reactive oxygen species (ROS) in the cytoplasm and nucleus and DNA double-strand breaks (DNA DSB) in the nucleus. PKA/PKB, ERK/MAPK, and ATM can phosphorylate CREB, which then translocates into the nucleus to bind CRE elements in order to express pro-survival proteins. ATM and DNA-PK can phosphorylate c-abl, which in turn phosphorylates YAP. Phosphorylated YAP acts together with p73 to stimulate expression of pro-apoptotic genes. ATM and DNA-PK can further induce Sp1, which can act pro-apoptotic by inducing p53 or pro-survival by regulating the DNA damage response and inducing DNA repair. IR-induced ROS can activate JNK to phosphorylate the AP-1 complex, thereby initiating DNA binding to TRE genes. Expression of TRE genes leads to induction of DNA repair and promotion of cell-cycle progression.

The *tyrosine kinase c-abl* is a functional analogous to p53 in regulation of programmed cell death and DNA repair (21) interacting with p53 indirectly through modification of upstream regulators [homeodomain-interacting protein kinase 2 (HIPK2)] (22). C-abl is the ubiquitously expressed product of the cellular homolog of the transforming gene of Abelson murine leukemia virus (v-abl) shuttling between cytoplasm and nucleus of the cell (21, 23). Cytoplasmic c-abl is assumed to function in association with the F-actin cytoskeleton while nuclear c-abl participates in cell-cycle regulation, DDR, and apoptosis (23). Sparsely ionizing leads to an activation of c-abl (21, 24, 25) *via* phosphorylation by ATM at Ser-465 (26) and by DNA-dependent protein kinase (DNA-PK) (21, 23). It can function as a negative regulator of DNA repair progression, inhibiting DSB re-joining and downregulating γH2AX, decreasing the recruitment of DNA repair factors to the damage site (21), or c-abl can phosphorylate DNA PK and Rad51 to abolish their binding to DNA, thereby impeding their function (21, 25). It can also promote apoptosis with a direct influence on the p73-dependent DDR by phosphorylating the YES-associated protein (YAP). Upon phosphorylation, YAP acts together with p73 on pro-apoptotic gene targets (Figure 2) (21, 22).

In response to densely IR, c-abl has been surmised to partake in a p53-independent induction of apoptosis. In the model described, c-abl activates caspase 9 *via* phosphorylation at Tyr 153, initiating the cleavage of caspase 3 as a point of no return in apoptosis induction (27).

In summary, a potential role in the cellular radiation response can be attributed to c-abl as mediator of apoptosis and coordinator of DNA repair. Albeit greater focus on densely IR, such as carbon therapy, has to be introduced to fully conclude its role for this radiation quality.

## Nuclear Factor κB

Although several genes induced by IR are p53-regulated, the majority are p53-independent (28–31), with the transcription factor NF-κB playing a contributing role (29, 31).

**Figure 3:**
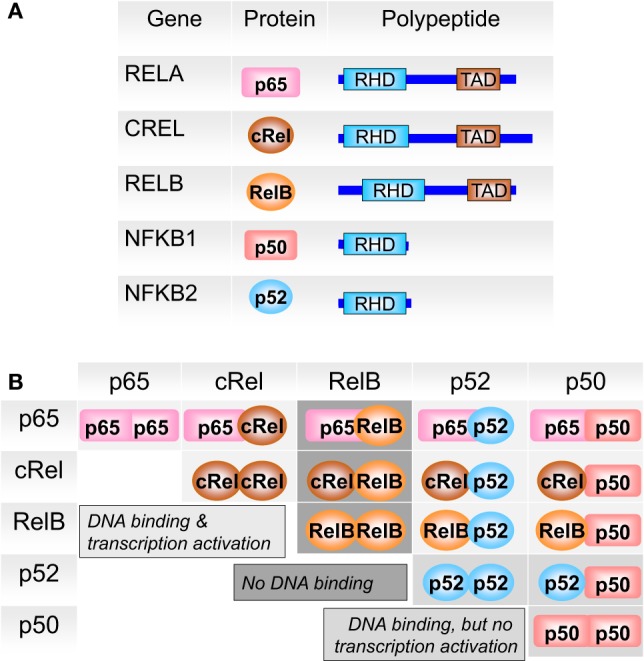
**The members of the NF-κB family**. **(A)** NF-κB subunits each contain a Rel homology domain (RHD) for dimerization and DNA binding. p65 (RelA), c-Rel, and RelB bear transcriptional activation domains (TAD). **(B)** The 5 NF-κB monomers can associate to 15 potential dimers. Of these, nine can bind DNA and activate gene transcription (light gray), three (the p50 or p52 only containing dimers) bind DNA but do not activate transcription (medium gray), and three do not bind DNA (dark gray). Adapted from O’Dea and Hoffmann (43).

*NF-*κ*B/Rel proteins* comprise a family of structurally related eukaryotic transcription factors that are involved in the control of a large number of cellular and organismal processes, such as immune system development and performance, inflammation, developmental processes, cellular growth, and apoptosis (32–35). Homo- or heterodimers of NF-κB1 (p50/p105), NF-κB2 (p52/p100), RelA (p65), RelB, or c-Rel (Figure 3) can be activated in response to hundreds of agents (36–38) and thereby modulate environment-induced gene expression. Besides immune modulating agents and pathogen-derived agents (lipopolysaccharides), a variety of other cellular stress factors are able to induce this pathway, such as cytokines, phorbol esters, viruses, ultraviolet (UV) radiation, reactive oxygen species (ROS), necrotic cell products, growth factor depletion, hypoxia, heat shock, and IR (38–41).

**Figure 4:**
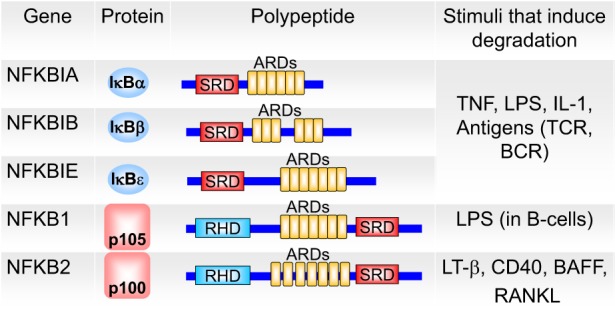
**The members of the inhibitor of NF-κB (IκB) family**. IκB proteins contain ankyrin repeat domains (ARDs) and signal response domains (SRDs) and are degraded in response to different signals (BCR, B-cell receptor; TCR, T-cell receptor; LPS, lipopolysaccharide; LT-β, lymphotoxin-β; BAFF, B-cell-activating factor; RANKL, receptor activator of NF-κB ligand). The ARDs on p105 and p100 (which are proteolytically processed to p50 and p52 NF-κB monomers, respectively) can act to self-inhibit p50 and p52. p100 can also form a multimeric complex in which it can inhibit other latent NF-κB dimers. Adapted from O’Dea and Hoffmann (43).

In the inactive state, NF-κB is retained in the cytoplasm by the inhibitory *I*κ*B proteins* (Figure 4), which controls nuclear translocation of NF-κB by masking its NLS (42, 43). *I*κ*B proteins* bind through their ankyrin repeat domain (ARD) to NF-κB. In their free state, IκB proteins are unstable and rapidly degraded, while binding to NF-κB strongly increases their stability (43). The three (NFκBIA, NFκBIB, and NFκBIE) genes code for the canonical IκB proteins, IκBα, IκBβ, and IκBϵ (Figure 4) (43). The p50:p65 heterodimer is mainly bound by IκBα. p105 and p100 proteins, which are involved in the alternative NF-κB pathway, contain the inhibitory part already in their C-terminal region in addition to the NF-κB part in the N-terminal half. Two novel IκBs (IκBζ and BCL-3) were described. BCL-3 is a non-inhibiting IκB family member that acts as transcriptional co-activator for p50:p50 and p52:p52 homodimers (44). Further novel atypical IκB proteins were recently reviewed by Arnemann et al. (45).

Upon activation, IκBα can be degraded by several proteases (46) and the released NF-κB translocates to the nucleus and binds to κB or κB-like DNA motifs [NF-κB response elements, NREs with the consensus sequence GGGRNNN(N)YCC]^1^ initiating gene transcription. NREs have been identified in the promoter or enhancer regions of more than 200 genes, including a number of IκBs, growth factors, proinflammatory cytokines (TNF-α, IL-1, IL-6) and enzymes (cyclooxygenase-2, COX-2), chemokines (IL-8/CXCL8; monocyte chemotactic cytokine 1, MCP-1/CCL2), angiogenic factors (vascular endothelial growth factor, VEGF), degradative enzymes (matrix metalloproteinases, MMPs), and adhesion molecules (intercellular adhesion molecule-1, ICAM-1; vascular cell adhesion molecule-1, VCAM-1; E-selectin). These target genes are involved in inflammation, innate immune responses, angiogenesis, tumor progression, and metastasis in various cancers and fibrosis (39, 44, 47, 48). NF-κB regulated expression of cytokines and extracellular matrix proteases after heavy ion exposure might contribute to extracellular matrix remodeling (49). Activation of NF-κB by high radiation doses (1–10 Gy) could contribute to the inflammatory response observed, e.g., in the developing brain after radiation exposure (50).

In addition, NF-κB also regulates the expression of many genes whose products are involved in the control of cell proliferation and cell death (51). In a cell culture model, it has been found that ATM plays a role in sustained activation of NF-κB in response to DNA DSB (52, 53), probably by its PI-3-kinase-like activity (54). Activation of the NF-κB pathway does not only protect cells from apoptosis after treatment with various genotoxic agents *via* expression of anti-apoptotic proteins, such as Bcl-2, GADD45β, TRAF-1, TRAF-2, cIAP-1, and cIAP-2 (55), but also gives transformed cells a growth and survival advantage and further renders tumor cells therapy resistant (56). NF-κB acts also as a transcriptional enhancer for the protective enzyme manganese superoxide dismutase (Mn-SOD) and might thereby contribute to therapy resistance. NF-κB also enhances the expression of degradative enzymes supporting the idea that it makes a major contribution to tumor progression and metastasis in various cancers (48). Therefore, NF-κB was identified quite early as potential target of innovative cancer therapies (57). Due to this anti-apoptotic effects NF-κB activation, it is an important stress response that may modulate the outcome of chemotherapy- and radiotherapy-induced toxicity.

For cells of the immune system, a misdirection of the NF-κB correlated process that normally creates immunoglobulin diversity might result in increased survivability of cells with oncogenic chromosomal translocations that prevent apoptosis and promote proliferation of pre-malignant cells. Constitutive activity of NF-κB or its over-expression has been reported for many human cancer cells (including breast cancer, colon cancer, prostate cancer, and lymphoid cancers) and can cause malignant changes in lymphoid cells in tissue culture.

NF-κB–Rel complexes can be activated and be functional in three different subpathways in different cells and tissues: the canonical or classical pathway, the alternative or non-canonical pathway, and the genotoxic stress-induced pathway (58–60).

In the *canonical or classical pathway*, the signals for activation of NF-κB are generated by cytokines, e.g., TNF-α or IL-1, by growth factors, by ligands of toll-like receptors (TLRs) or by antigens, which bind to the T-cell receptor (TCR) or the B-cell receptor (BCR). The NF-κB is mostly composed of p50:p65 and p50:c-Rel dimers.

**Figure 5:**
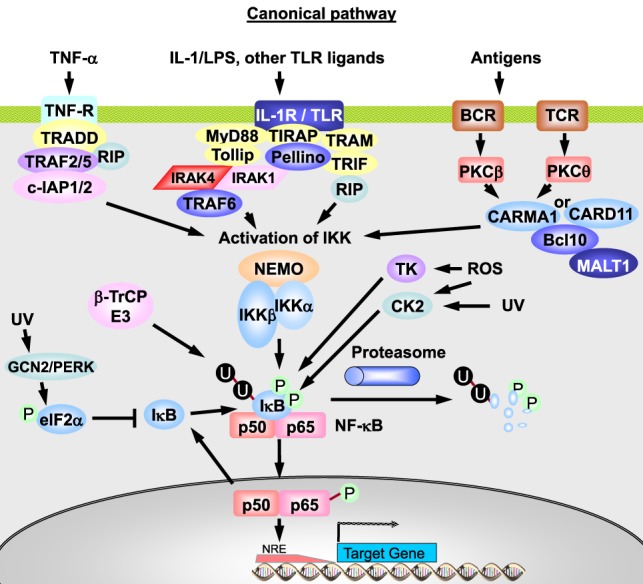
**The canonical or classical NF-κB pathway**. The binding of TNF-α to TNF-R leads to a rapid recruitment of TRADD, RIP1, TRAF2, TRAF5, c-IAP1, and c-IAP2. Formation of this complex triggers TRAF2/5 and c-IAP1/2 to catalyze polyubiquitination of RIP1 and autoubiquitination of TRAF2 and/or c-IAP1 (not shown). Modified RIP1 then recruits the TAK1/TAB1/TAB2 (only TAK1 shown) and IKKα/IKKβ/NEMO complexes, leading to TAK1 activation and TAK1-mediated activation of IKKβ. Upon IL-1 stimulation of IL-1R, proteins, such as MyD88, Tollip, IRAK-1, and IRAK-4, are recruited, leading to IRAK1/4-dependent binding of TRAF6 and Pellino. TRAF6 then undergoes autoubiquitination, whereas Pellino catalyzes IRAK1 ubiquitination. Ubiquitinated TRAF6 in turn serves as a platform to recruit the TAK1/TAB1/TAB2 complex, resulting in TAK1 activation and finally IKKβ activation. TLR signaling can be MyD88 dependent or independent through TRAM, TRIF, and RIP. Activated IKK then phosphorylates IκBα, resulting in its ubiquitination and degradation. This IκBα degradation allows p50:p65 dimer to translocate to the nucleus and activate the expression of genes involved in inflammation, innate immunity, and cell survival. Ultraviolet (UV) irradiation reduces IκB levels *via* activation of GCN2 or PERK, which phosphorylate the initiation factor elF2α, and *via* casein kinase 2 (CK2) and thymidine kinase (TK). Phosphorylated elF2α blocks IκB synthesis. The BCR and TCR are expressed by B- and T-lymphocytes and do not act one the same cell. Adapted from O’Dea and Hoffmann (43) and Habelhah (44).

A central event in the pattern of NF-κB complex activation (Figure 5) is the activation of IκB kinase (IKK). This is achieved *via* a complex pathway involving several adaptor proteins, ubiquitin ligases, binding proteins, and kinases, such as receptor-interacting protein 1 (RIP1) and TNF-R-associated factor 2, 5, or 6 (TRAF2/5/6) (43, 44, 58–60), resulting in activation of IKK kinases (*IKK-K*). These kinases are responsible for phosphorylation of IKK and might be TGF-β-activated protein kinase 1 (TAK1) or MAPK kinase kinase 3 (MEKK3) after stimulation with TNF-α (44). The exact contribution of different kinases to IKK activation is not completely known, and redundancy in function may occur.

The *IKK* complex is composed of the two catalytic subunits, IKKα and IKKβ,^2^ and the regulatory subunit, IKKγ/NF-κB essential modulator (NEMO) (43). The activated IKK phosphorylates IκB at the serine residues 32 and 36 in the signal responsive domain and thereby targets IκB for ubiquitination (61). Phosphorylated IκB is polyubiquitinylated by the E3 ubiquitin ligase containing β-TrCP and subsequently degraded by the 26S proteasome (43, 58, 59). Alternatively, IκB can be phosphorylated at tyrosine 42, which has the potential to connect NF-κB directly to membrane receptor-associated tyrosine kinases (62).

Receptor signaling as described above in the canonical pathway is often dependent on the synthesis of autocrine factors, such as cytokines (64).

In response to TNF-α, IκBα is rapidly degraded, followed by NF-κB-dependent resynthesis. Persisting stimulation by binding of TNF-α to its receptor (TNF-R) results in cycles of IκBα degradation and resynthesis (43). After TNF-α stimulation, the deubiquitinases A20 (or TNF-α-induced protein 3, TNFAIP3) or CYLD (gene mutated in familial Cylindromatosis)^3^ limit NF-κB activation (44, 65).

Activation of NF-κB after antigen binding to the TCR or BCR is mediated *via* activation of a phospholipase, which produces diacylglycerol, the activator of protein kinases C (PKC). PKC phosphorylates caspase recruitment domain 11 (CARD11), which then recruits other adaptor proteins – BCL-10 and MALT1 forming the CBM complex^4^ with CARD11 in B-cells – leading finally to phosphorylation of IKKβ and ubiquitination of NEMO (Figure 5). The activated IKK complex phosphorylates IκB, leading to its degradation, as described above (65).

**Figure 6:**
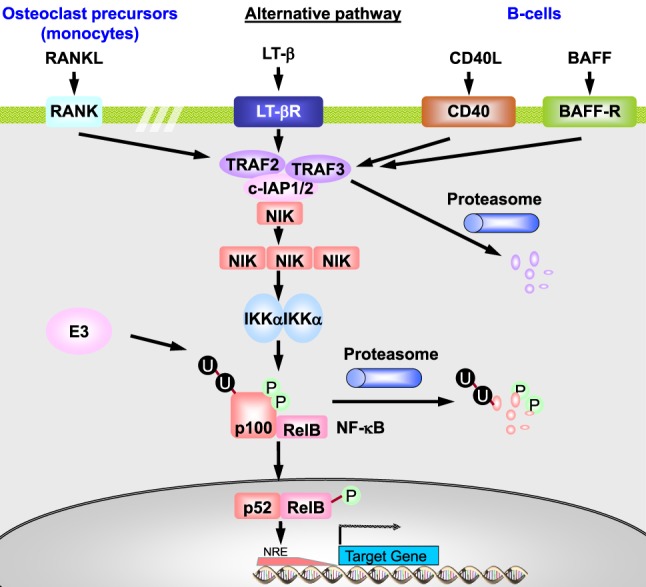
**The non-canonical or alternative NF-κB pathway**. In unstimulated cells, TRAF3 constitutively recruits NIK to the TRAF2–c-IAP1/2 complex, promoting c-IAP1/2-mediated K48-ubiquitination and degradation of NIK. Ligation of CD40 by CD40L leads to recruitment of the TRAF3–TRAF2–c-IAP1/2 complex to the receptor, where TRAF2 catalyzes polyubiquitination of c-IAP1/2. It thereby promotes ubiquitin E3 ligase activity of c-IAP1/2 toward TRAF3, leading to proteasomal degradation of the latter. As a result, NIK can no longer be recruited to the TRAF2–c-IAP1/2 complex. This leads to stabilization and accumulation of newly synthesized NIK and its activation presumably *via* autophosphorylation, resulting in activation of the IKKα homodimer. Activated IKKα then phosphorylates p100, leading to proteasome-mediated processing of p100 to p52. The p52:RelB heterodimer then translocates to the nucleus and regulates transcription of target genes. CD40L promotes antibody isotype switching in mature B-cells, RANKL initiates osteoclastogenesis from precursor cells, BAFF induces immune cell survival and proliferation of B-cells, and LT-β regulates lymph node development. The receptors represented here on one cell are therefore restricted to distinct cell types and usually do not act in parallel. Adapted from O’Dea and Hoffmann (43) and Habelhah (44).

The *alternative or non-canonical pathway* is involved in non-inflammatory signaling, e.g., in lymph node development and osteoclastogenesis (43). It starts at membrane receptors of the TNF-R superfamily with binding of B-cell activation factor (BAFF), lymphotoxin β (LTβ), CD40 ligand (CD40L), or receptor activator of NF-κB ligand (RANKL) (Figure 6). BAFF is critical for B-cell survival. LTβ is involved in lymph node development. CD40L has functions in the adaptive immune response, such as B-cell proliferation and differentiation, and immunoglobulin isotype switching. RANKL is essential for osteoclast differentiation from monocytes. Receptor–ligand binding results in activation of the IKKα-containing kinase complex by NF-κB-inducing kinase (*NIK*) and sometimes of the canonical IKKβ-containing complex (43, 44). TRAF2, c-IAP1, and c-IAP2 negatively regulate NIK *via* ubiquitination- and proteasome-dependent degradation (43). In unstimulated cells, NIK is marked for degradation by the TRAF2/c-IAP1/2 complex. After receptor binding, NIK is stabilized and forms trimers that activate the IKKα complex.

In the alternative pathway, inactive NF-κB consists of a p100:RelB heterodimer. p100 in the p100:RelB complex is phosphorylated by the activated IKKα, which results in polyubiquitination of p100 and proteolytic degradation of the NF-κB-inhibiting C-terminal region of p100, releasing p52 (43). The resulting p52:RelB dimer translocates to the nucleus where it initiates transcription of genes involved in lymphoid organogenesis, immune cell survival, proliferation and maturation, and osteoclastogenesis (44). It results in low level nuclear translocation of NF-κB for hours or days (43).

Several cross-talk mechanisms of inflammatory and developmental NF-κB signaling *via* the canonical and the alternative pathway were described (43). For example, developmental signals can also activate canonical p50:p65 dimers bound to a dimer of p100 (called IκBδ) (43). The activated NIK as part of the alternative pathway may amplify canonical IKK activation (43). CD40L can activate the canonical and the alternative NF-κB pathway (65).

Recently, a role for RelB was suggested in the therapy resistance of prostate cancer, which was explained by RelB-dependent induction of MnSOD (67).

DNA DSB in the cell nucleus can activate the *genotoxin-induced pathway* (68). Early studies supposed DNA-PK (69), PI3K, and MAPK (64) as mediators of radiation-induced NF-κB activation. In a cell culture model, it has been found that *ATM* plays a role in sustained activation of NF-κB in response to DNA DSB (52, 53), probably by its PI-3-kinase-like activity (54). Activation of NF-κB *via* this pathway after exposure to IR was described in detail in a recent review (70). Briefly, DNA damage initiates the SUMOylation of *NEMO* by the sumo ligase PIASy in a complex with PIDD and RIP1, fostering NEMO’s localization in the nucleus (43). This nuclear SUMOylated NEMO associates with ATM with the result of monoubiquitination of NEMO, which is the signal for its cytoplasmic export (43). SUMOylated NEMO in complex with ATM therefore represents the long searched nuclear to cytoplasmic shuttle of the NF-κB activating signal (68). The protein ELKS binds to the cytoplasmic ATM–NEMO complex, enabling ATM-dependent activation of the canonical IKK complex (43). As described for the canonical pathway, IKK activation leads to IκBα degradation and NF-κB activation. The canonical p50:p65 heterodimer initiates gene transcription in the nucleus.

The role of the NF-κB pathway in cellular radiosensitivity was addressed by several studies (6). In a human ovarian cancer cell line, a human breast cancer cell line, and a murine melanoma cell line, radiation-activated NF-κB protected the cells from radiation-induced apoptosis (71). Inhibition of NF-κB activity can be achieved by overexpression of dominant-negative, phosphorylation-defective IκB. This has been reported to enhance the radiosensitivity of human fibrosarcoma cells (72), xenografted fibrosarcomas in mice (73) and human brain tumor cells (61, 74, 75), and to influence X-ray-induced mutations and apoptosis in human malignant glioma cells (76). For low LET radiation, NF-κB inhibition increased radiosensitivity of many cancer cells (6).

As activation of the NF-κB pathway is supposed to play a role in the negative regulation of both death receptor- and stress-induced apoptosis (44), survival of cells with residual DNA damage might thus be favored. Also, NF-κB’s role in the deregulation of inflammatory responses contributes to its tumor-promoting and progression-favoring characteristics (44).

*Constitutive NF-*κ*B activation* is often found in human cancers, e.g., breast, thyroid, bladder, and colon cancer (56, 77–80). It is thought to be important for maintaining survival of the cancer cells and for angiogenesis or chemoresistance (43, 81). The mechanisms that lead to constitutively activated NF-κB (82) and its critical role in tumor progression are currently only partly understood for some tumors. Mutations of the NFKBIA gene, which encodes IκBα or alterations of its expression level, might be an explanation for the increased NF-κB activity in tumors. In a recent study analyzing 790 human glioblastomas, deletion or low expression of NFKBIA was associated with unfavorable outcomes (83), possibly resulting from uncontrolled NF-κB activity. In 37.5% of patients with Hodgkin lymphoma, mutations in the NFKBIA gene in the tumor cells were detected (84). Lake et al. (85) found NFKBIA mutations in 3 of 20 Hodgkin lymphoma patients (15%). In addition, a NFKBIA polymorphism (A to G variation, rs696 in the 3′ UTR)^5^ was associated with colorectal cancer risk and poor treatment prognosis (86). In human adult T-cell leukemia or lymphoma associated with human T-cell leukemia virus type I, activation of the NF-κB pathway by the virus protein Tax *via* the canonical and the alternative pathway seems to be involved in the transformation process (47). Another mechanism of constitutive NF-κB activation was described in malignant melanoma cells: an elevated endogenous ROS production resulted in constitutive NF-κB translocation to the nucleus (87).

## Nuclear Erythroid-Derived 2-Related Factor 2

Nuclear erythroid-derived 2 (NF-E2)-related factor 2 (Nrf2) that binds the antioxidant DNA response element (ARE) to induce cellular antioxidant defense systems was shown to be activated 5 days after irradiation (88). Nrf2 was identified in studies investigating the activation of detoxifying enzymes in the presence of electrophilic chemicals, such as ROS. It belongs to the cap “n” collar (CNC) family of basic leucine zipper (bZIP) transcription factors. In vertebrates, they include the p45–NF-E2 factors and the NF-E2-related factors Nrf1, Nrf2, and Nrf3. In fact, Nrf2 was first identified as a homolog of NF-E2 and was found to interact with NF-E2-binding sites (89). The natural repressor protein of Nrf2 is Kelch-like associated ECH-associated protein 1 (Keap1), also called inhibitor of Nrf2 (INrf2).

Whereas NF-E2 was found in erythroid cells, Nrf1 and Nrf2 expression was observed in many tissues (90). In humans, the Nrf2 gene is located on chromosome 2q31 and in mice on chromosome 2. Target genes of Nrf2 contain a specific binding region, the ARE or electrophilic response element (EpRE). The ARE consensus sequence is TGA(G/C)NNNGC (89). Target genes of Nrf2 include detoxifying enzymes and antioxidative enzymes, such as glutathione-*S*-transferase, superoxide dismutase (SOD), or NADPH reductase.

The Nrf2 gene consists of five exons and four introns and its promoter region contains two ARE sequences, indicating that Nrf2 controls to some extent its own expression. ARE regions are also found in the promoter regions of Keap1 and the small Maf protein MafG (91–93). The presence of an ARE sequence in the Keap1 gene suggests an auto-regulatory feedback loop between Nrf2 and Keap1 (93).

**Figure 7:**
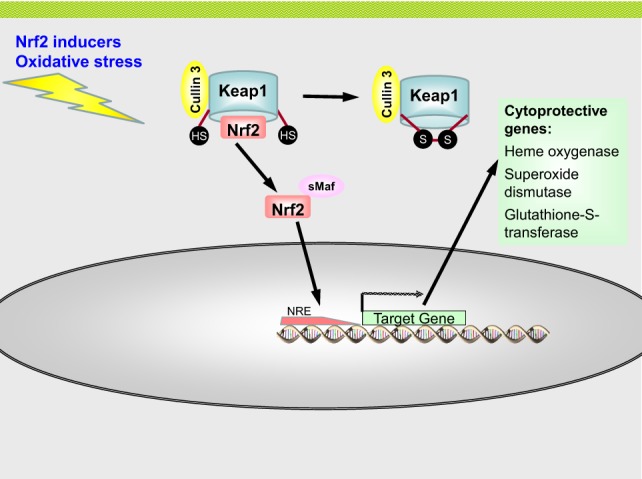
**The Nrf2–ARE pathway**. Nrf2 is sequestered in the cytoplasm by Keap1 and targeted for ubiquitination by Cullin 3 and proteasomal degradation. Under conditions of oxidative stress or by chemical activators the thiol groups of cysteine residues of Keap1 are oxidized. This leads to the formation of disulfide bridges, which changes the conformation of Keap1 which is unable to bind Nrf2 now. Nrf2 is no released from Keap1 and translocates to the nucleus by forming heterodimers with sMaf proteins. In the nucleus, the Nrf2–sMaf complex binds to antioxidant responsive element (ARE) sequence in the promoter region of Nrf2 target genes, leading to the expression of antioxidative enzymes, such as heme oxygenase, superoxide dismutase, and glutathione-*S*-transferase.

The Nrf2 protein consists of 605 amino acids in humans and 597 amino acids in mice (90, 94), and is subdivided into six domains, which are evolutionary highly conserved and are termed Nrf2–ECH homology domains, abbreviated Neh1–6. They play important roles in binding to DNA, activation and inactivation of Nrf2. Protein structure, genetics regulation, and history of discovery of Nrf2 are summarized in the in-depth reviews of Baird and Dinova-Kostova (94), Ramkissoon et al. (90), and Morita and Motohashi (89). Baird and Dinova-Kostova discuss several possible activation and regulation mechanisms of Nrf2, i.e., the sequester and release model, which was used in Figure 7, the “dissociation of Keap1 and Cullin 3 model,” the “hinge and latch model,” the “Keap1 nucleocytoplasmic shuttling model,” and the “ubiquitination of Keap1 model,” as well as some evidence suggesting that Nrf2 directly senses stressors (94). In this review, the “sequester and release model” will be featured (Figure 7), but it should be mentioned that it is a significant simplification of the actual mechanism underlying Nrf2 regulation within cells. What is most important from a radiation biological point of view about Nrf2 and its repressor Keap1, it is the fact that the Nrf2–ARE pathway is redox sensitive. IR induces the formation of free radicals, mainly due to radiolysis of water molecules within cells.

Reactive oxygen species form adducts with DNA, proteins, carbohydrates, and lipids. Cells are naturally exposed to ROS due to metabolic processes and by other environmental cues. Cells possess different defense mechanisms to maintain their redox equilibrium by increasing the expression of antioxidative enzymes. The activity of Nrf2 depends on the ROS level within a cell.

Nuclear erythroid-derived 2-related factor 2 is repressed by the protein Keap1 under physiological conditions. Keap1 consists of 624 amino acids and is rich in cysteine residues (25 in mice and 27 in humans) (94). Keap1 binds to Nrf2 and sequesters it in the cytoplasm and acts as a scaffold for the Cullin 3–Rbx1-E3 ubiquitin ligase, thereby promoting the proteasomal degradation of Nrf2. Under basal conditions, the half-life period of Nrf2 is thus only 10–30 min (90). As mentioned above, Keap1 possesses many cysteine residues, whose thiol groups are oxidized by ROS, leading to the formation of disulfide bridges. This changes the conformation of Keap1, releasing Nrf2 from its binding to Keap1. Nrf2 then translocates to the nucleus together with co-transcriptional factors, such as small Mafs (also belonging to the class of bZip transcription factors). Nrf2 forms heterodimers with the small Mafs, which then bind to ARE regions.

Nuclear erythroid-derived 2-related factor 2 has many interaction partners: apart from the small Maf proteins (MafG, MafF, and MafK), another cotranscription factor is the CREB-binding protein (CBP). Nrf2 activity is downregulated by nuclear import of Keap1 where it binds Nrf2 and exports it back to the cytoplasm. Furthermore, the kinase Fyn phosphorylates the tyrosine residue 568 of Nrf2, which is a signal for the nuclear export of Nrf2 (90). Of importance is also the interaction of Nrf2 and NF-κB. NF-κB competes for CBP binding and inhibits Maf kinases (95). Keap1 was shown to contribute to the degradation of the NF-κB subunit p65 (96). ERK1/2-dependent pathways were suggested to mediate Nrf2 activation by low-dose γ-irradiation (97).

McDonald et al. (88) investigated the impact of γ-irradiation on Nrf2 activity in mouse embryonic fibroblasts derived from Nrf2 wt and knockout (ko) mice. They analyzed the expression of Nrf2 target genes by quantitative real time RT-PCR (RT-qPCR) and Nrf2 activity by a luciferase reporter system. The reporter system consisted of a plasmid where a firefly luciferase gene was fused with a promoter containing the ARE. No significant increase of either target gene expression or Nrf2 activity could be observed in this cell line 24 h after single exposure to even a high dose of up to 10 Gy of γ-radiation. Yet, 5 days after irradiation, Nrf2 activity markedly increased and this response persisted up to 8 and 15 days after irradiation. By shifting to a fractional irradiation over a time period of 5 days, a significant increase in Nrf2 target gene expression and increased reporter gene expression was observed 3 h after the end of fractionated irradiation. Additionally, they observed no effect on cellular survival of inducing Nrf2 prior to irradiation in murine fibroblasts, lymphocytes, and dendritic cells by using different chemical activators that activate Nrf2 in a brief time period of a few hours. However, immortalized fibroblasts derived from Nrf2-ko mice were more susceptible to irradiation than immortalized fibroblasts derived from Nrf2 wt mice. The increase of ROS inside cells was delayed as well with a response after 5 days. The level of ROS as measured by the fluorescence dye 2′,7′-dichlorofluorescin diacetate (DCF-DA) only increased 5 days after irradiation and was higher in Nrf2-ko fibroblasts, which moreover showed a higher basal ROS level. McDonald et al. (88) concluded from these results that Nrf2 is important for regulating long-term radiation effects and functions as a buffer system for maintaining the redox equilibrium in cells; a function than cannot be altered by exogenous chemical factors, but it is important for long-term cellular survival after radiation exposure. They speculated that long-term radiation effects may be related to an altered mitochondrial function, which results an increased production of ROS. Datta et al. (98) confirmed this for cells of the small intestine by exposing mice to γ-irradiation and accelerated iron (Fe-56, 1000 MeV/n; LET 148 keV/μm) ions. They observed a more potent induction of oxidative stress, DNA damage, and apoptosis in the small intestine of mice, which were exposed to energetic iron ions compared to mice that were γ-irradiated. Interestingly, they also observed long-term changes in the metabolism and gene expression in the intestinal tissue of mice up to 1 year after irradiation: mitochondrial function was altered and the level of ROS produced by mitochondrial metabolism increased as well as expression of NADPH oxidase, resulting in persistent elevated ROS level in the small intestine. Datta et al. (98) suspect that the induction of long-term effects after irradiation is important for the evaluation of chronic or late radiation effects.

Jayakumar et al. (99) found a connection between differences in Nrf2 expression and radiation resistance in the two different prostate cancer cell lines PC3 and DU145. Both cell lines were exposed to γ-radiation and Nrf2 content was measured prior to and after irradiation. Both cell lines differed in Nrf2 expression under basal conditions. It was found that cellular survival was higher after irradiation in the cell line DU145, which showed a higher basal Nrf2 activity. Furthermore, the level of basal and induced ROS after irradiation was higher in PC3 cells, which in contrast to DU145 exhibited a lower basal activity of Nrf2. In both cell lines, Nrf2 protein content increased after irradiation, whereas the protein level of Keap1 was reduced. Also, the expression of antioxidant enzymes increased after irradiation in both cell lines, but this response was significantly stronger in the DU145 cell line. Cellular survival was reduced in both cell lines and especially in the cell line with low basal Nrf2 expression PC3, after exposure to the chemical Nrf2 inhibitor retinoic acid. This outcome was enhanced when Nrf2 was knocked down on transcript level by transfecting both cell lines with small interfering RNA (siRNA) against Nrf2. These findings indicate that Nrf2 is important for cellular survival after exposure to IR and that radiation resistance of different cell lines, especially cancer cell lines depends upon differences in basal Nrf2 activity and upregulation of Nrf2. Examining the expression of Nrf2 in tumor tissue may therefore be important for the planning and predicting the outcome of therapeutic irradiation. Furthermore, as predominantly antioxidant enzymes were upregulated, there seems to be an association between oxidative stress and cellular survival after irradiation. Independently from radiation biological considerations, Wang et al. (100) showed that Nrf2 is antagonized by retinoic acid receptor α (RARα) in the small intestine of mice after treatment with retinoic acid, this opens the possibility to modulate Nrf2 activity chemically. Mathew et al. (101) showed that the Nrf2 activator sulforaphane (which also shows anti-cancerous effects) protects fibroblasts against IR.

In a similar setting, Patwardhan et al. (102) showed that T cell lymphoma EL-4 cells exhibit a high basal Nrf2 activity, show lower ROS levels than non-tumorigenic cells and that enhanced radiation resistance is linked to higher Nrf2 activity. siRNA against Nrf2 or treatment with all-*trans* retinoic acid (ATRA also called Tretinoin as a pharmaceutical) enhanced radiosensitivity of EL-4 cells.

Apart from IR, such as X-rays and γ-radiation, Nrf2 was also shown to be activated by UV radiation (103). Dermal fibroblasts showed an increased accumulation of Nrf2 in the nucleus after exposure to UV-A irradiation, but not after UV-B irradiation. Moreover, fibroblasts derived from Nrf2-ko mice exhibited a 1.7-fold increase in apoptosis after UV-A exposure compared to fibroblasts derived from wt mouse. The opposite effect was observed in Keap1-ko fibroblasts; here, the apoptosis rate was half of the rate observed in fibroblasts isolated from wt mice.

Nuclear erythroid-derived 2-related factor 2 also seems to be important for the function adult stem cells. Hochmuth et al. (104) showed that inactivation of the Nrf2 analog CncC in *Drosophila* was required for the maturation of intestinal stem cells. CncC is constitutively active in intestinal stem cells and its inactivation by Keap1 is a signal for intestinal stem cell proliferation. They observed that increased ROS levels lead to increased proliferation of intestinal stem cells, a similar effect could be observed by knocking down CncC with siRNA. This work indicates that the activation pattern of CncC is reversed in stem cells compared with differentiated cells. Whereas in differentiated cells ROS activate CncC or Nrf2, in intestinal stem cells CncC is inactivated by ROS to allow for an increased proliferation of intestinal stem cells to renew the intestinal tissue. This seems to imply an activation mode of Nrf2 that depends on cell type and developmental stage. How oxidative stress inactivates Nrf2 in intestinal stem cells remains to be investigated. Tsai et al. (105) could confirm the role of Nrf2 in controlling stem cell proliferation. They confirmed the findings of Hochmuth et al. (104) in hematopoietic stem cells. Reduced Nrf2 activity or lack of Nrf2 led to hyperproliferation of stem cells and progenitor cells. This was accompanied by a reduced self-renewal of hematopoietic stem cells and reduction of quiescence. Nrf2 proved to be a crucial factor in the regulation and function of hematopoietic stem cells. Whereas both studies did not involve radiation, Kim et al. (106) showed that Nrf2 contributes strongly to the survival of hematopoietic stem cells in a mouse model of total body γ-irradiation. Nrf2-ko mice had a lower survival and a higher impairment of hematopoietic function compared to Nrf2 wt mice. Furthermore, activation of Nrf2 prior to irradiation increased overall survival of mice after TBI. Additionally, hematopoiesis was increased in mice, which were treated with the Nrf2 activator 2-trifluoromethyl-2-methoxychalone (TMC).

Recently, Large et al. (107) investigated the role of Nrf2 in the low-dose response of ECs. Surprisingly, they found a decreased expression of Nrf2 after low-dose X-irradiation (0.5 Gy) in EA.hy926 ECs and primary human dermal microvascular ECs (HMVEC), additionally the DNA binding of Nrf2 was lowered. The expression of Nrf2 target genes and the activity of Nrf2 itself followed a non-linear pattern of expression, meaning that the observed level of mRNA increased and decreased interchangeably with increasing doses (0, 0.3, 0.5, 0.7, and 1 Gy). In all cases, the expression of glutathione peroxidase (GPx) and SOD as well as the Nrf2 binding activity were lowest after 0.5 Gy X-irradiation. This finding is interesting, as it indicates that at least in these cell lines low-dose irradiation may induce an unusual pattern of Nrf2 expression and activity and that certain doses may decrease Nrf2 activity. As they treated cells prior to irradiation with TNF-α to simulate inflammation, there might be an involvement of NF-κB, as TNF-α activates it, in the expression and activity pattern of Nrf2.

Relatively little is known about the role of Nrf2 in the cellular radiation response to heavy ion irradiation. Quite recently, Xie et al. (108) showed that curcumin (an Nrf2 activator) reduces cognitive impairment in mice after exposure carbon ions (4 Gy). They observed an upregulation of Nrf2 and Nrf2 downstream genes, NAD(P)H quinine oxidoreductase 1 (NQO1), heme oxygenase-1 (HO-1), and γ-glutamyl cysteine synthetase (γ-GCS) in the brain tissue of mice that were treated with curcumin. Kim et al. (109) found increased survival of colonic epithelial cells after exposure to iron ions (Fe-56) when Nrf2 was activated prior to radiation exposure. They focused on the interaction of Nrf2 and p53 binding protein 1 (p53BP1), as p53BP1 contains three ARE sequences. Activation of Nrf2 in colon epithelial cells prior to irradiation reduced the frequency of G1 and S/G2 chromosome aberrations. In general, the DDR was enhanced by Nrf2 activation.

The response of Nrf2 to radiation may be an important factor in tumor therapy. Mutations in Nrf2 and Keap1 are known to occur in various cancer cell lines and to confer protection to cancer cell lines toward chemo- or radiotherapy. Some cancer cell lines possess a high basal activity of Nrf2, which increases their resistance against radiation (110). In general, Nrf2 is accumulated in tumors and high levels of Nrf2 expression lead to a poor prognosis for cancer patients (111). In a lung cancer study, samples were taken from 178 lung squamous cell carcinomas (SqCCs) (112, 113) and, apart from 53BP1, most mutations were found in three genes associated with the Nrf2–ARE pathway, NFE2L2 (the gene name of Nrf2), Keap1, and Cul3 (the gene name of Cullin 3). Increased resistance to radiation therapy was linked to a high level of Nrf2.

Further studies of the function and activity of Nrf2 after exposure of cells to heavy ions, especially carbon ions, may give hints how to improve radiation therapy and will be of significance for the planning of long-term human space missions.

## Role of Other Transcription Factors in the Cellular Radiation Response

In addition to the two key transcription factors in the cellular damage response, p53 and NF-κB, and the oxidative stress-induced Nrf2, several other transcription factors are activated in response to IR exposure (Figure 2). ATM-mediated phosphorylation of c-Abl appears to increase the transcription of stress response genes *via* activation of Jun kinase (15). Also, other MAPK pathways are activated, resulting, e.g., in formation of the transcription factor activated protein 1 (AP-1), which controls proliferation, senescence, differentiation, and apoptosis *via* growth factors (114). Cataldi et al. (115) propose a role for the transcription factor cyclic adenosine monophosphate (cAMP)-responsive element-binding protein (CREB) in survival responses of Jurkat T-cells after exposure to IR. The transcription factor Sp1, which is involved in cell differentiation, cell growth, apoptosis, immune responses, response to DNA damage, and chromatin remodeling, is rapidly phosphorylated in response to DNA damage, but this phosphorylation did not affect its transcriptional activity (116). The colocalization of phosphorylated Sp1 with activated ATM kinase in nuclear foci was interpreted as a sign for a role of Sp1 in DNA repair (116). A colocalization in nuclear foci was also observed for the forkhead box-O 3 (FOXO-3) transcription factor that is involved in cell-cycle control (117).

### cAMP-Responsive Element-Binding Protein

cAMP-responsive element-binding protein (CREB) is a 43-kDa bZIP nuclear transcription factor involved in cAMP signaling (115). Its activation *via* phosphorylation leads to binding of the transcription factor to the cAMP-responsive element (CRE), a highly conserved sequence, inducing transcription of target genes for several cellular functions, including regulation of apoptosis and proliferation (118, 119). CREB phosphorylation is cell type and stimulus dependent. It is therefore possible for a wide range of molecules to activate the transcription factor. PKA, PKB, and ERK/MAPK phosphorylate CREB at Serine 133 (119, 120), whereas ATR and ATM phosphorylate the factor at Serine 111 and 121, respectively, in response to IR and oxidative stress (121, 122).

Sparsely IR is able to increase the binding of CREB to its consensus sequence (123). Furthermore, γ-irradiation has been shown to induce phosphorylation of CREB, thereby activating it (115, 119). Radiation-induced activation of CREB is connected with survival, as its target genes promote cellular proliferation, such as cyclin A, cyclin D1, proliferating cell nuclear antigen (PCNA), c-fos, and COX-2. In CREB knockdown studies, survival decreased (124). Co-incidental with CREB activation is low caspase-3 activity and a lack of Bax and Bcl2 level difference, further supporting an anti-apoptotic role (119).

Although an increase of ERK/MAPK expression after irradiation with carbon ions (125) and α-particles (126) was shown, no connection to CREB activation has been made. Therapeutic high LET studies lost their focus on CREB as survival factor, but it can be said that with the upcoming trend of therapeutic irradiation using heavy ions, CREB should be considered as means to increase radiosensitivity in tumor cells.

### Activated Protein 1

Activated protein 1 (AP-1) is a transcription factor formed by homo- or heterodimerization of proteins of the Jun family (c-Jun, JunB, and JunD) or the Fos family (c-Fos, FosB, Fra-1, and Fra-2). Such dimers (AP-1 complexes) are able to recognize AP-1 binding sites containing the 12-*O*-tetradecanoylphorbol-13-acetate (TPA) response element (TRE), its DNA target sequence (127). C-jun regulates the expression of cyclin D1, a promoter of cell-cycle progression into G1-phase (128, 129). In addition to genes regulating cell-cycle progression, AP-1 controls its own expression, having a TRE in the promoter region of the c-jun gene (130).

The transcriptional activity of c-Jun can be enhanced by phosphorylation of Serine 63 and 73 by JNK. It has been shown that c-Jun is phosphorylated by JNK in the nucleus after γ-irradiation (Figure 2). Furthermore, the same study showed increased DNA-binding activity of AP-1 in response to sparsely IR with AP-1 complexes containing c-Jun as well as JunD and JunB (131). X-radiation has also been shown to increase DNA-binding activity of AP-1 (132). Exposure to an 18 MeV electron beam (4 Gy) activated AP-1 in HepG2 cells (133).

Elevated gene expression of c-jun and protein expression of AP-1-associated factors (c-jun, c-fos, Fra1, and JNK2) was observed in response to α-particles exposure (134, 135). Binding activity of AP-1 increased after irradiation with very low doses of α-particles (6 mGy). This binding was inhibited by SOD indicating a response of AP-1 to oxidative stress (126). Iron ion (Fe-56, 1000 MeV/n, LET 148 keV/μm) irradiation has been associated with proliferation of intestinal epithelium cells, connecting irradiation-induced oxidative stress with activation AP-1 (98).

Reactive oxygen species and other radiation-released free radicals can stimulate JNK and AP-1 activity (23), therefore promoting cell-cycle progression, although influence in apoptosis induction has also been reported (136). Additionally, the radiation-induced activation of AP-1 was also correlated to increased levels of glutamylcysteine synthetase, which is directly associated with synthesis of glutathione, a cellular radical scavenger (133). Therefore, AP-1 might be relevant in high LET radiation therapy by enhancing the cellular defense against ROS and regulation the cellular apoptotic response to radiation.

### Specificity Protein 1

The Sp1 belongs to the specificity protein/Krüppel-like factor (Sp/XKLF) family of transcription factors that contain 3 conserved Cys2His2 zinc fingers for DNA binding (137). Loss of these zinc fingers abolishes not only DNA-binding capacity but also nuclear translocation (138). Sp1 is ubiquitously expressed in all mammalian cells and regulates cellular functions, such as apoptosis, cell-cycle progression, growth/proliferation, and metabolism (137, 139). The fate of Sp1 differs greatly depending on its posttranslational modification. Many different proteins modify Sp1 through all stages of the cell cycle *via* SUMOylation, glycosylation, ubiquitination, acetylation, or phosphorylation. Overexpression of Sp1 regulates apoptosis in a p53-dependent manner after suppression of cell growth (137).

ATM can activate Sp1 *via* phosphorylation in response to X-rays and H_2_O_2_ (140), which is recruited to DNA DSB and can promote repair in a non-transcriptional manner (141). Sp1 acts also in a transcriptional manner upon IR, as it is associated with coordination of cellular response after treatment with γ-rays, activated by DNA-PK through phosphorylation (142). Furthermore, an increased nuclear expression of Sp1 has been observed for irradiation with 20 Gy X-rays (143) as well as increased binding activity upon γ-irradiation (123, 144).

Microarray gene expression experiments assume activation of Sp1 in cells irradiated with α-particles (LET 123 keV/μm), and its involvement in subsequent cellular responses of directly and indirectly exposed (bystander) cells (145). The Sp1-dependent gene expression profile included up- and downregulation of 16 and 6 genes, respectively, in cells directly hit by α-particles, while upregulation of a smaller subset (10 genes) dominated in bystander cells (145). As with sparsely IR, Sp1 could act in a more administrative manner upon high LET radiation, as DNA-PK is strongly activated after carbon and iron ion exposure (146).

Sp1 has various roles in the DDR, ranging from orchestrating the response, to actively regulating apoptosis or repair. For cancer treatment with carbon ions, Sp1 is well worth investigating, as there are not many distinct approaches to study this versatile transcription factor in context of high LET particle irradiation.

### Early Growth Response 1

The transcription factor early growth response 1 (EGR-1) is a member of the EGR family and is suggested to act as anti-proliferative signal for tumor cells, as well as an apoptotic enhancer (147). It has been shown to be activated by X- (147) and γ-rays (148). Radiation-induced activation of EGR-1 is associated with ROS (148). Upon irradiation, EGR-1 can act p53-independently as mediator for TNF-α-induced apoptosis (147). Gene expression of EGR-1 has been found to be increased also in response to irradiation with α-particles (134). The role of EGR-1 in the cellular radiation response is pro-apoptotic and in light of heavy ion radiation therapy, it would be instructive to know the extent of its pro-apoptotic capabilities upon high LET irradiation.

## Influence of Linear Energy Transfer on Transcription Factor Activation

The cellular response to high LET radiation shows quantitative and in some aspects qualitative differences compared to the low LET radiation response. For different radiation types, the biological effects, observed at the same absorbed dose, depend on their quality (sparsely or densely IR). Comparison of the biological effects of different radiation qualities is usually being performed in terms of relative biological effectiveness (RBE).^6^ In radiotherapy, the RBE is not only of highest interest for cell killing but also for late effects such as cancerogenesis (149). For the various biological endpoints, the RBE can depend on many factors, such as LET, dose rate, dose fractionation, radiation dose, and type of the irradiated cells or tissues.

One of the earliest systematic studies of the dependence of RBE on LET showed that the RBE reached a maximum at an LET of 100–200 keV/μm for survival of human kidney T1 cells after irradiation with deuterium $\left({\;}_{1}^{2}H\right)$ and α-particles (151–153). Thereafter, an LET–RBE function was determined for many biological endpoints and reached a maximum at with an LET from 90 to 200 keV/μm (154–157). In these studies, the RBE for mutation induction was higher compared to inactivation for all examined LETs (154). In HEK cells, the maximal RBE for reproductive cell death was 2.5 (158). For LETs above 900 keV/μm, RBE values for reproductive cell death dropped to 1 or below 1. Stoll et al. (159) also found an RBE for inactivation by high LET lead ions (>10,000 keV/μm) far below 1 and for nickel ions (>1000 keV/μm) around 1. In a human neuronal progenitor cell line (Ntera2), the RBE_max_ for apoptosis 48 h after iron ion exposure (1 GeV/n) was at least 3.4 (160). The RBE for induction of double-strand breaks was determined to be 1.8 for iron ions compared to X-rays, as detected by immunostaining of γ-H2AX 0.5 h after radiation exposure (161), or by pulsed-field gel electrophoresis (162) or other methods such as alkaline elution (163). For α-particles (LET 27–124 keV/μm), it ranged between 1.2 and 1.4 (164).

For improvement of cancer therapy, studies with several cancer cells and with various heavy ions, especially carbon ions, had been performed. In a microarray analysis of oral SqCC cells, 84 genes were identified that were modulated by carbon and neon ion (LET ~75 keV/μm) irradiation at all doses (1, 4, and 7 Gy) (165). Among these genes, three genes (TGFBR2, SMURF2, and BMP7) were found to be involved in the transforming growth factor β signaling pathway and two genes (CCND1 and E2F3) in the cell-cycle G1/S checkpoint regulation pathway. The relevance of these results for normal tissues cells or non-cancer cell lines has to be determined. In normal skin tissue, low doses (0.01 and 1 Gy) of IR resulted in transient alterations in the expression of genes involved in DNA and tissue remodeling, cell-cycle transition, and inflammation (TNF, interleukins) (166), suggesting an involvement of the NF-κB pathway, the main inflammatory pathway, in the cellular response to IR. As exposure to accelerated argon ions (95 MeV/n Ar, LET 271 keV/μm) resulted in strong activation of NF-κB in human cells (167), the RBE for NF-κB activation by heavy ions of different LET was determined (158). NF-κB-dependent gene induction after exposure to heavy ions was detected in stably transfected human 293 reporter cells. For comparison, cells were exposed to 150 kV X-rays. The maximal biologic effect ranged between 70 and 300 keV/μm. Argon ions (271 keV/μm) had the maximal potency (RBE ~9) to activate NF-κB-dependent gene expression in HEK cells. The effect of carbon ions was less pronounced and comparable the activation observed after X-ray exposure (168). Inhibition of ATM resulted in complete abolishment of NF-κB activation by X-rays and heavy ions. Therefore, NF-κB activation in response to heavy ions is ATM dependent and seems to be mediated by a nuclear signal from the damaged DNA as described for the genotoxin-induced NF-κB subpathway.

Assuming that NF-κB activation promotes survival, it can be hypothesized that the extreme capacity of energetic heavy ions in the LET range of 70–300 keV/μm to activate NF-κB’s transcriptional effects might be responsible for the lower relative effectiveness in cell killing observed in this range. Above 300 keV/μm, the overkill effect (meaning that with further increase of the deposited energy in a small volume of the cell no more biologically relevant damages can be caused) possibly results in a decrease of the RBE.

Other groups report NF-κB translocation after exposure of normal human monocytes (MM6 cells) to 0.7 Gy of ^56^Fe ions using a DNA-binding assay (169). This clearly indicates that high LET iron ion exposure induces rapid and persistent NF-κB activation. This activation of NF-κB was shown to be mediated through phosphorylation of IκBα and the subsequent proteasome-dependent degradation pathway. The iron study only revealed binding of NF-κB to its consensus sequence of 5′-GGGGACTTTCC-3′, and not transcriptional activation.

Scarce LET dependence data exist for p53 expression in human neuronal progenitor cells (160). Screening of gene expression in the nematode *Caenorhabditis elegans* suggests an LET dependence or track structure dependence of the gene expression changes (170). In human lens epithelial cells, transcription and translation of CDKN1A [p21^CIP1/WAF1^] are both temporally regulated after exposure to 4 Gy of high-energy accelerated iron-ion beams (~150 keV/μm) as well as to protons (~1 keV/μm) and X-rays, whereby the magnitude and kinetics of the expression enhancement seem to depend on the LET of the radiation (171).

## Conclusion

Increased understanding of signaling pathways leading to transcription factor activation or inhibition in response to high LET radiation exposure will help to identify and make use of new targets for radiosensitization of tumor tissue and/or increasing radioresistance of surrounding normal tissue. The question which transcription factor offers a suitable target for charged particle cancer therapy is still open, as very few studies on transcription factor activation by carbon ions in tumor cells were performed. Also, not in all studies clinically relevant doses were applied, and extrapolation of the effects from high to lower doses is not constructive when the dose–response curves are unknown.

Although the role of p53 seems to be quite clear in low LET radiation therapy with increased radiosensitivity in case of functionality, this is not yet the case for charged particle therapy. Some studies suggest p53-independent cell killing by high LET which is a large advantage for treatment of tumors with p53 mutations. More studies with different cancer cell types are required. Concerning surrounding tissues, p53 inhibition might prevent precipitous apoptosis in apoptosis prone tissues. In tissues where p53-induced cell-cycle arrest dominates, p53 inhibition might impede this protective pathway and have detrimental effects.

The anti-apoptotic effects of NF-κB could support tumor cell survival during chemo- or radiotherapy; therefore, NF-κB is an interesting target for combined cancer therapies including carbon ion therapy. In several tumor cell types, inhibition of NF-κB resulted in radiosensitization. Activation of NF-κB in the normal tissue might not only limit detrimental effects by cell killing but also promote inflammation.

The activity of Nrf2 after irradiation seems to follow a complicated pattern, i.e., different time scales seem to be involved, and most striking is the occurrence of long-term effects in fibroblasts and intestinal epithelial cells. In the case of intestinal epithelial cells, the occurrence of long-term oxidative stress points to an inefficient activation of Nrf2 or a reduced oxidative stress response. It would be interesting to investigate this further. Of particular interest would be to compare differentiated cells with tissue-specific stem cells after irradiation; as in the case of intestinal stem cells, some studies suggest that oxidative stress inhibits the action of Nrf2 and that here might be differences between fully differentiated cells and stem cells in the regulation of Nrf2. Another open question is how to modulate the Nrf2 response in healthy tissue for radiation protection to reduce the side effects of radiation therapy and to mitigate radiation effects in spaces in case of manned missions. Modulating the elevated level of Nrf2 activity in cancer cells may be beneficial for cancer therapy. As described above, Nrf2 is upregulated in many cancer tissues and a high level of Nrf2 expression corresponds to a poor prognosis for patients. In this sense, Nrf2 can serve as an indicator for the outcomes of cancer radio- and chemotherapy. So far, relatively little is known about chemicals that may inhibit Nrf2 directly (apart from retinoic acid) or upregulate Keap1, research in this direction may lead to the discovery of novel drug candidates supplementing radiation therapy. Using siRNA for therapeutic means may also be an option for those cancers that show a high expression and activity of Nrf2.

CREB, AP-1, Sp1, and EGR-1 (or up- or downstream events in these pathways) were activated by low doses of high LET α-particles and the first three were also shown to be involved in the cellular response to carbon and/or iron ions. For low LET radiation, many studies suggest a subordinate importance of these factors in cellular radiation responses compared to p53, NF-κB, and Nrf2. Nevertheless, we discuss how modification of these transcription factors may influence therapy results.

CREB itself is a factor inducing cellular survival, activated by phosphorylation due to (among others) ATM. Amorino et al. (124) detected a decreased survival of CREB-ko cells after IR exposure compared to wt cells. As also high LET irradiation stimulates ATM kinase activity, which in turn can activate CREB, a potential therapy approach is to inhibit phosphorylation of CREB or its binding to DNA in the vicinity of cancerous tissue. This can be accomplished through siRNA or other CREB-inhibiting substances, which are then applied directly to target tissue (in case of superficial tumors) or transported to the tumor *via* homing probes (in case of hard-to-reach tumors). After reducing the survivability of the tumor in such a way, radiation-induced apoptosis *via* p53-dependent and -independent mechanisms can fight the tumor more effectively.

This approach bears the problem that, with drug-induced inhibition of CREB in tumor vicinity, also non-transformed tissue may be affected and therefore more susceptible for radiation-induced cell death. In this case, the high fidelity of dose deposition in heavy ion therapy may prove advantageous.

AP-1 is a sensory factor for oxidative stress and is activated by ROS and other free radicals besides IR (low LET like X- and γ-rays as well as high LET α particles). This activation can lead to apoptosis; therefore, a supplementary approach in heavy ion therapy would be to enrich AP-1 in tumor tissue, delivered as mentioned above, so that more AP-1 is activated by high LET radiation and apoptosis is induced more effectively in tumor cells. C-abl is a negative regulator of γH2AX and can induce apoptosis in a p53-independent manner. Therefore, a supplementary strategy for heavy ion therapy is again enrichment of the protein to increase apoptotic induction and weaken repair of damaged DNA in tumor cells.

Sp1 is, besides other functions, a regulator for apoptosis and the orchestration of the DDR and can initiate repair of DNA damages. An obvious strategy to reinforce high LET radiation effects would be inhibition of Sp1. However, the transcription factor assumes many various roles within the cell, so that manipulation of Sp1 might result in unwanted side effects. Genes regulated by Sp1 might be better targets for combating tumors and their expression in tumor cells in response to heavy ion irradiation should be analyzed.

Upon activation, the transcription factor EGR-1 has various pro-apoptotic and anti-proliferative effects in tumors. Its activation has been demonstrated so far for low LET irradiation and high LET α-particles. Before considering it as target in charged particle therapy, its response to carbon ion exposure should be determined. If high LET radiation induces pro-apoptotic effects in tumors cells *via* EGR-1 as well, an artificial enrichment of the factor could be beneficial.

These various strategies are attuned to particular effects of each respective transcription factor and could be amplified *via* combination. However, cross connectivity of these factors with other signaling pathways may exert considerable influence on the therapeutic result. Furthermore, radiation-induced bystander effects, inter- and intracellular modifications in response to various signaling factors, are strongly to be considered for both tumor and healthy tissue. It is undoubtedly certain that the more we know about pathway connections among themselves, especially in respect to heavy ion therapy, the better are chances to develop more effective strategies to fight tumors.

In summary, above mentioned transcription factors and associated proteins are involved in a wide spectrum of cellular functions upon treatment with IR, ranging from regulation of cell cycle, DNA repair, cell proliferation, differentiation, adhesion, migration, and apoptosis to immune responses including inflammation. All factors show increased activity and/or expression for low and (as far as tested) high LET irradiation and are involved in the cellular radiation response.

## Author Contributions

CH had the idea for this review, designed it and contributed the introduction, the NF-κB chapter, the NF-κB Figures 3–6, and the conclusion, redesigned Figures 1, 2, 7, inserted the references, corrected and edited all other parts, especially the conclusion, and did the revision according to the reviewers’ comments. LS wrote the p53 chapter, contributed the p53 input for Table 1, and designed Figure 1. BH wrote the Nrf2 chapter, contributed the Nrf2 input for Table 1, and designed Figure 7. SD wrote the chapter “other transcription factors,” contributed to Table 1, and invented Figure 2. CB-K contributed to the idea and design of the review and critically reviewed and corrected the manuscript.

## Conflict of Interest Statement

The authors declare that the research was conducted in the absence of any commercial or financial relationships that could be construed as a potential conflict of interest.

## Footnotes

^1^N, any nucleotide, R, purine, and Y, pyrimidine.

^2^“The commonly used anti-inflammatory drugs aspirin and sodium salicylate exert their effects in part by acting as competitive inhibitors of the ATP-binding site of IKKβ” (63).

^3^Mutations in the CYLD gene are very rare, affected patients develop benign tumors on the skin due to increased cell growth mediated by overactive NF-κB (66).

^4^Abnormal BCR signaling *via* CD79 and the CBM complex was observed in B-cell lymphomas (44, 65). Elevated MALT1 expression was also observed in lymphomas of mucosal-associated lymphoid tissue and might be explained in early stages by constant antigenic stimulation, and later by chromosomal translocations that position the MALT1 gene under the control of heterologous promoters (38).

^5^UTR, untranslated region at the 3′ end of the mRNA. The reference single nucleotide polymorphism (SNP) Cluster Report rs696 is available in the National Center for Biotechnology Information (NCBI) SNP database (http://www.ncbi.nlm.nih.gov).

^6^The absorbed dose of a test radiation necessary to induce a defined biological endpoint and a defined severity of this endpoint (survival, cell cycle arrest, mutagenesis, chromosome aberrations, tumor induction in laboratory animals, and others) is compared to the dose of a reference radiation needed for induction of the same biological effect (150). Sparsely ionizing radiation such as γ-rays or X-rays is often used as reference radiation. In some cases, protons are applied as reference. The RBE is calculated according to the following formula:

$$\mathrm{RBE}=\frac{\mathrm{Absorbed}\;\mathrm{dose}\;\mathrm{of}\;\mathrm{reference}\;\mathrm{radiation}\;\mathrm{inducing}\;\mathrm{biologic}\;\mathrm{effect}\;\mathrm{(Gy)}}{\mathrm{Absorbed}\;\mathrm{dose}\;\mathrm{of}\;\mathrm{test}\;\mathrm{radiation}\;\mathrm{inducing}\;\mathrm{bioloogic}\;\mathrm{effect}\;\mathrm{(Gy)}}$$
